# Designing an exercise intervention for adult survivors of childhood cancers

**DOI:** 10.1186/s12885-020-07763-8

**Published:** 2021-01-04

**Authors:** Denise Rokitka, Jennifer Heffler, Michael Zevon, Caleb Kitcho, Jennifer Schweitzer, Elisa M. Rodriguez, Martin C. Mahoney

**Affiliations:** 1 Department of Pediatrics, Roswell Park Comprehensive Cancer Center, Buffalo, New York 14263 USA; 2 Department of Cancer Prevention and Control, Roswell Park Comprehensive Cancer Center, Buffalo, New York 14263 USA; 3 Department of Internal Medicine and Department of Health Behavior, Roswell Park Comprehensive Cancer Center, Buffalo, New York 14263 USA

**Keywords:** Cancer survivors, Child/adolescent cancers, Quality of life, Physical activity

## Abstract

**Background:**

This study examined current physical activity levels and preferences for exercise settings and activities among adult survivors of childhood cancers as a strategy to inform the feasibility and design of such programs.

**Methods:**

A mixed-methods design was used to investigate current activity levels as well as barriers to and preferences for physical activity among 20 adult survivors of pediatric cancer.

**Results:**

One-half of participants reported engaging in regular physical activity, although the frequency, intensity, and duration varied. Overall, 17 of the 20 participants (85%) stated they would be interested in participating in a structured exercise intervention, and they expressed a strong interest in walking (76%), bicycling (53%), and weight training (53%). Common barriers to participation in a potential structured exercise program were insufficient time, current health issues, and program location/distance. Nearly all participants agreed that information on nutrition and diet should be included as part of an exercise intervention.

**Conclusions:**

These findings will help inform the design and implementation of future exercise programs to enhance physical activity among this high-risk group of cancer survivors.

**Supplementary Information:**

The online version contains supplementary material available at 10.1186/s12885-020-07763-8.

## Background

Advancements in cancer detection and treatments mean that approximately 80% of all patients diagnosed with childhood cancers will survive for at least 5 years. However, cancer survivors often experience immediate and long-term treatment-related complications across a range of functional domains and exhibit an increased prevalence of chronic health conditions (e.g., cardiac, pulmonary, endocrine, reproductive, neurocognitive) [1]. A recent analysis by Hudson et al. of the St. Jude Life Cohort found that by age 45 years, 95.5% of survivors had at least one chronic health condition compared to 30.6% of the general U.S. population [2].

To promote good health and reduce the risk of multiple chronic diseases, the U.S Department of Health and Human Services recommends at least 150 min total of moderate-intensity, or 75 min total each week of vigorous-intensity aerobic activity, or a combination of moderate and intense activity [3]. Although one report has suggested that survivors of childhood cancer engage in physical activity at a rate comparable to the general population [1, 2], more recent studies have reported that survivors are less likely to meet physical activity guidelines recommended by the Centers for Disease Control [4–7].

Persistent treatment-related side effects, as well as fatigue, reduced muscle strength, and emotional difficulties, have the potential to complicate or hinder healthy lifestyles for cancer survivors [8, 9]. Additional barriers to wellness activities among childhood cancer survivors include lack of resources, negative thoughts and feelings toward a healthy lifestyle, and negative environmental and social influences [10].

The purpose of this study was to better understand the specific needs and preferences of adult survivors of childhood cancer, and to inform the design of future intervention studies, by examining survivors’ current self-reported health and physical activity levels, interest in structured exercise programs, and their specific preferences for survivor-focused interventions.

## Methods

### Participants and recruitment

Inclusion criteria for participants were survivors between the ages of 18 to 60 years old who had been diagnosed with cancer at age ≤ 21 years old, were > 5 years past the date of their original diagnosis, had completed all active cancer therapy, had an anticipated life expectancy of more than 12 months, and were followed at Roswell Park Comprehensive Cancer Center (RPCCC). Batches of letters were mailed to randomly selected survivors in our cohort requesting participation until target accrual of responses was achieved. Interested patients were prescreened by phone to confirm eligibility prior to consenting. Participants were offered the option to complete the structured interview either in person or over the phone. Consent was obtained in person by all participants prior to enrollment on the study. Approval for this study was granted by the Institutional Review Board (IRB) at RPCCC.

### Design

This study used a mixed methods research design that incorporated both quantitative and qualitative data collection.

### Data collection

Demographic and clinical information was obtained from the medical record. Structured interview questions were developed by the study team and were informed by the existing literature. Questionnaire is provided as an additional file 1: Structured Intervew, and is not a validated questionnaire. These questions explored current level of physical activity (e.g., duration [in minutes], frequency [number of days per week], types of activities) as well as interest in and preferences for future involvement in structured exercise programs. Interviews were conducted by research staff and were recorded and transcribed. Interviews lasted approximately 20–25 min.

### Analyses

Audio-recorded structured interviews were transcribed and reviewed for accuracy. The transcripts were entered into the qualitative data analysis software package NVivo (QSR International, Victoria, Australia) and organized by nodes which corresponded to interview questions. Two members of the research team then individually open-coded to distinguish concepts and categories within nodes, to identify preliminary findings and to assure thematic saturation. Discrepancies were resolved by reviewing the original responses and discussing applicable concepts or categories to reach agreement. A table of final concepts was made to organize results, allow for final review of the areas assessed, and to reveal any potential gaps in the data.

Quantitative data were reported using ranges, means, medians, and standard deviations for continuous variables, and as frequencies and relative frequencies for categorical variables. Pearson chi-square and Mann-Whitney tests were used to compare responses by category. All analyses were performed using SPSS version 21 (© IBM, Cary, NC).

## Results

### Quantitative findings

As presented in Table 1, the 20 participants included 11 women (55%) and nine men (45%), ranging in age from 21 to 52 years old (mean age = 35). Most of the participants were white (85%), and the majority were employed outside the home (85%). More than half (55%) reported that they had obtained a bachelor’s degree or higher, though all participants had completed at least some college.

**Table 1:** Selected Demographics and Clinical Characteristics of Study Participants Compared to Institutional Cohort

	Participants (***n***=20)	Total Cohort (***N***=1061)	
	**n (%)**	**n (%)**	**p- value**
**Current Age (in years)**			0.723
≤ 29	7 (35.0)	318 (30.0)	
30–39	6 (30.0)	27 (26.0)	
40–60	7 (35.0)	467 (44.0)	
**Race**			0.731
White	19 (95%)	960 (90.5)	
Other	1 (5%)	101 (9.6)	
**Sex**			0.323
Male	9 (45.0)	595 (56.1)	
Female	11 (55.0)	466 (43.9)	
**Age at Diagnosis**			0.267
0–4	6 (30.0)	236 (22.0)	
5–9	4 (20.0)	229 (21.0)	
10–15	1 (5%)	237 (22.3)	
16–21	9 (45%)	359 (33.8)	
**Years Since Diagnosis**			0.155
5–9	–	68 (6.4)	
0–19	7 (35.0)	269 (25.4)	
20–29	8 (40.0)	266 (25.1)	
30+	5 (25.0)	458 (43.2)	
**Type of Cancer**			0.460
Leukemia	8 (40.0)	281 (26.5)	
Lymphoma	6 (30.0)	323 (30.4)	
CNS	1 (5.0)	79 (7.4)	
Sarcoma	4 (20.0)	179 (16.9)	
Other	1 (5.0)	199 (18.8)	

Seven participants (35%) described their current overall level of physical activity as low, nine (45%) reported moderate overall levels of physical activity, and four (20%) described their overall physical activity level as high. Minutes of physical activity per day ranged from 10 to 360 min, with a median of about 98 min per day. Higher levels of physical activity generally appeared to be reflective of more physically demanding occupations. Level of physical activity did not differ significantly by sex, current age group, marital status, or level of education. However, a history of radiation therapy was associated with lower median minutes of activity per day (Mann Whitney U = 16.5, *p* = 0.010).

Fourteen participants (70%) indicated that they believed exercise had the potential to decrease risk of cancer and that they had discussed the benefits of exercise with their physician. Sixteen respondents (80%) indicated that they were concerned about their current body weight, and body mass index (BMI) levels were consistent with this concern as ten (50%) of the participants were overweight (BMI 25–29.9) and five (25%) were obese (BMI ≥30).

Nine participants (45%) currently had a membership to a gym or health club, and two (10%) had previously paid for a trainer or health professional to assist them with diet and/or exercise. 45% (9 persons) were currently using a diet/fitness app or a wearable fitness tracker and three others (15%) indicated that they would use one if it were relevant to them. Slightly less than one-half of the sample (45%) reported using a diet or exercise app on their smartphone. Thirteen participants (65%) expressed a desire to learn more about the benefits associated with exercise and nutrition.

Nine respondents (45%) reported that they were currently participating in a structured exercise program.

### Interest in an exercise intervention program

Seventeen participants (85%) indicated that they would be interested in a structured exercise program that would be of no cost to them, including eight who stated a willingness to commit to a “long term/continuous” program. Preferred times for a potential exercise program varied: six (30%) stated they would prefer to exercise in the morning; ten (50%) said they would prefer afternoons or evenings, and one (5%) did not state a preference (3 participants were uninterested in an exercise program).

When asked how many days per week they would be available to participate in a structured exercise program, responses ranged from 1 to 5 days; though slightly under half of this subsample—eight people (47%)—stated that they would be willing to participate 3 days per week. Fifteen of the 17 respondents interested in an exercise program (88%) stated that they would be willing to spend 60 min at each exercise session. Participants identified several preferred exercise locations: “private gym” (9/17, 53%), “at home” (6/17, 35%), “at the cancer center” (7/17, 41%), “at the university” (6/17, 35%) and “other location” in the community (7/17, 41%). (Percentages do not add to 100% because multiple responses were permitted).

The majority of participants (13/17, 76%) indicated no preference for exercise done either independently or in a group with other cancer survivors. Nearly all participants (16/17, 94%) strongly endorsed an exercise program that combined both aerobic and resistance training. Most commonly endorsed activities included walking (12/17, 71%), bicycling (9/17, 53%), and weight training (9/17, 53%) were the most commonly endorsed activities. (Percentages do not add to 100% because multiple responses were permitted).

All respondents were asked to identify two or three factors that could be an obstacle or barrier to their participation in a structured exercise program. Responses generally fell into three categories—time/schedule, distance/location, and physical limitations. Sixteen participants (80%) identified a lack of time and scheduling conflicts—including work schedules and possible conflicts with family time and childcare—as major barriers. A majority of respondents (60%) also expressed concern that “distance from the exercise location” could be a potential barrier, particularly in the event of inclement weather. Physical limitations such as “not feeling well enough” or “issues with my leg” were noted by 10%. Participants did not report emotional issues or lack of motivation as barriers to physical activity.

Reported barriers did not differ by demographics, treatment variables or current level of physical activity. Finally, almost all participants (95%) felt that information on nutrition and diet should be included as part of an exercise intervention, and 85% indicated that they would attend educational lectures on diet and nutrition.

#### Qualitative findings

Analysis of participants’ responses yielded both motivators for and barriers to remaining physically active.

### Motivators

#### Physical and mental health

Six respondents (30%) reported they were motivated to exercise because of the general benefits to physical and mental health. A survivor of embryonal rhabdomyosarcoma replied, “I like feeling energetic, feeling sore the next day, feeling exhausted.” Another survivor of osteosarcoma expressed, “I just want to feel better.” Two other survivors said they exercised to keep their body weight down and to reduce stress.

Interestingly only two of these six participants specifically referenced their previous history of cancer as motivating their commitment to regular exercise. A neuroblastoma survivor stated, “I think having a past of being sick and knowing that exercise helps is definitely a major motivator.” This sentiment was echoed by another participant who had undergone treatment for Hodgkin lymphoma: “Because of all the treatments I went through… I want to keep my body healthy.”

#### Interpersonal relationships

Three participants identified interpersonal relationships as their impetus for physical activity. One participant explained, “My boyfriend is a personal trainer so that helps a lot.” Two women responded that they were motivated to exercise because of their children: “Just kind of feeling better and setting a good example for my kids,” and “My daughter [motivates me].”

### Barriers

The 11 participants not currently participating in a structured exercise program were asked about obstacles to their involvement, yielding responses which fell into one of two categories: time constraints and current health issues.

#### Lack of time

Eight participants (40%) reported that it was difficult to regularly find time to exercise, usually because of work schedules. A survivor of acute lymphoblastic leukemia explained, “Probably the biggest thing is time, that’s pretty much the main thing.” Another respondent agreed: “Busy scheduling, until recently I have been exclusively working overnights.”

#### Current health problems

Three respondents said that health conditions limited their participation in structured exercise. A survivor of a germ cell tumor said, “I think I have a circulatory problem. I get sore when I am on my feet for a while.” Another survivor noted, “I would say my legs, I really can’t bend them as well anymore and I still keep getting cramps in my feet” and another explained, “But the thing is, I had osteosarcoma so my leg can’t stand a lot of…can’t run, because it’s like, a lot of pounding and stuff like that.”

## Discussion

Preventive care is a critical element of health care among the growing cohort of adult survivors of pediatric cancer; consequently, the focus of the current study was to better understand levels of interest in as well as potential barriers to and preferences for a physical activity/exercise intervention. Current level of physical activity was included to understand actual levels of exercise among participants, as well as their interest in a potential exercise intervention. This data was collected to inform the design of a survivor- informed exercise intervention to enhance feasibility and relevance to the target population of adult survivors of childhood cancers.

Published studies support the efficacy of physical activity in reducing the risk of both disease recurrence and secondary complications among cancer survivors [11–13]. Recent research has also examined the feasibility and efficacy of a variety of physical activity or exercise interventions [14], yet persistent challenges remain to both recruiting and retaining participants in such studies.

Several studies have identified common facilitators of exercise and healthy diet that include social and cognitive motivators (e.g., the development of goals and routines), positive social relationships, wanting to gain a sense of control over physical, emotional and mental wellbeing, being equipped with “tools” for health behavior such as access to gyms, and general education on healthy diet and food preparation [9, 10, 15]. This aligns with results of the present study where cancer survivors expressed an interest in earlier and more comprehensive education about late effects, as well as a desire for better management of treatment-related physical and emotional stressors that may impede motivation. Consistent with observations in the present study, survivors have also sought improved social supports, possibly in the form of survivor exercise groups, and/or programs better tailored and more accommodating to survivor specific needs [16].

It was encouraging to note that 85% of participants indicated that they would be interested in participating in a structured exercise program. Although factors such as “time” and “distance” were commonly noted as potential barriers to participation, these barriers to participation did not differ by gender, age, treatment-related factors, or current activity level. These barriers, while challenging, are generally consistent with the prior literature in cancer survivors and in the general population [16, 17]. A survey of 144 survivors of pediatric cancers between the ages of 13–35 found that common barriers to exercise included being too tired (57%), a lack of time (53%), and not having a gym membership (48%) [16].

Study participants also expressed detailed preferences supporting the development of a more tailored, survivor-focused intervention. Contrary to a previous study [17], participants did not specifically endorse a program that involved exercising with other survivors, which may be related to potential concerns about time and location. Preferences for the location of a potential exercise program were diverse, which may reflect an underlying challenge for creating a “catch all” or even a “catch most” program. Of note, participants endorsed one supervised session weekly with the remaining sessions completed by them independently.

Based upon these responses, we envision that a survivor-informed exercise intervention would consist of one sixty-minute exercise session per week, with guidance for additional physical activity sessions to be completed at home. Furthermore, use of fitness trackers and diet and exercise apps for smartphones may be reasonable alternatives or additions to an exercise program that would facilitate interactions among participants. Researchers have begun to integrate wearable fitness technology and activity trackers in their study designs of exercise programs for cancer survivors [18, 19]. In a home-based, randomized controlled trial assessing the efficacy of a physical activity intervention for sedentary breast cancer survivors, 86 women were assigned to a physical activity group or a control group. After randomization, women in the physical activity group were initially counseled in person on how to do moderate-intensity exercises and each received a journal to log their physical activity and a Digiwalker (a pedometer) to wear while they exercised. Every week for 12 weeks, each woman in the physical activity group received exercise tip sheets as well as physical activity counseling via telephone. Analyses conducted after treatment revealed that women in the intervention group had engaged in significantly more physical activity than the control group. They also had significant improvements in fatigue reduction [20]. This type of approach may prove to be a pragmatic solution which could provide participants with desired structure and oversight while also addressing stated barriers.

Survivor input regarding a potential exercise intervention is generally consistent with other published studies. In one study, researchers implemented a 10-week intervention among adult survivors of pediatric cancers which encouraged a general increase in physical activity (e.g., walking, gardening, and housework) for participants to complete independently [18]. Participants reported their progress to a counselor via telephone at three, six, and 9 weeks, and used a pedometer to measure steps. Subjects were recruited from a long-term follow-up clinic: 67 met inclusion criteria, 21 refused to participate, 46 enrolled, and eight withdrew from the study. However, the 38 participants who completed the intervention showed improvements in both activity level and fatigue. Similarly, a home-based intervention among childhood cancer survivors treated with anthracyclines found improved cardiac function after 3 months of structured aerobic and weight bearing exercise [14]. Though successfully implemented, these interventions occurred over on limited time interval and did not track long-term participation and outcomes.

While not a primary focus of the current study, we were surprised to note that participants consistently expressed interest in further education on diet and nutrition. Although the majority of participants reported having previously discussed this topic with a clinician, a more detailed and comprehensive educational component appears warranted. This finding was generally consistent with recent literature suggesting that survivors desired more education on health behaviors, particularly as they relate to late effects of treatment [21]. One meta-analysis observed unhealthy diets across all age groups of cancer survivors, noting low fruit and vegetable intake, low calcium intake, and high fat intake [22]. While these behaviors are also observed in the general population, given long-term effects from cancer treatment and increased risks for future complications such as metabolic syndrome, heart failure, and type 2 Diabetes among survivors, diet and nutrition education could be of great importance. The proposed schedule of a single supervised session weekly would permit incorporating a focused educational diet/nutrition module and provide regular opportunity for follow-up and feedback by attendees.

Limitations of the study include a small number of participants recruited from a single cancer center and self-reported exercise and activity levels. In addition, similar to many studies, physical activity was assessed retrospectively by querying subjects as opposed to prospective assessment. Because participants were asked to project their interest in a future program, there is the possibility that their interest is over-estimated. However, information solicited from survivors on how to best construct this future program is nonetheless valuable.

Strengths of the current study include the relevant and unique insights regarding current levels of physical activity and desired features of an exercise intervention (e.g., time, place, frequency, component activities) as expressed by adult survivors of childhood cancers. Further, the current study provides specific program preferences expressed by survivors supporting the development of a more tailored approach to an exercise intervention, as well as the inclusion of other components such as diet and nutrition, which may prove to drive participant interest in a more holistic “wellness program”.

While multiple studies have established the importance of physical activity among childhood cancer survivors, the feasibility and implementation of programs for this purpose remains a challenge. Understanding current practices, barriers to participation, and survivor preferences will help to improve the quality, relevance and implementation of future exercise programs.

## Conclusions

Childhood cancer survivors are at risk for long term health complications that can be mitigated with attention to nutrition and exercise. We demonstrate that adult survivors of childhood cancers are interested in both physical activity interventions and nutrition education, however these programs need to be accessible and convenient to participants.

## Supplementary Information

**Additional file 1.** Structured Interview

## Data Availability

All data generated or analyzed during this study are included in this published article.

## Abbreviations

RPCCC: Roswell Park Comprehensive Cancer Center
IRB: Institutional Review Board
BMI: Body Mass Index
