# Supplementary material for: Designing an exercise intervention for adult survivors of childhood cancers
Source: BMC Cancer. 2021 Jan 4;21:1. doi: 10.1186/s12885-020-07763-8 (PMC7784286; doi:10.1186/s12885-020-07763-8)
Supplement: Supplementary file 1 — Additional file 1. Structured Interview [file 12885_2020_7763_MOESM1_ESM.docx]

**Structured interview: Exploring Barriers to Physical Activity among Adult Survivors of Childhood Cancers**

Interviewer:

Date:

Start Time:

End Time:

**Health/Physical Activity:**

1. How would you rate your overall health?

A) Excellent

B) Very good

C) Good

D) Fair

E) Poor

2. How would you describe your overall level of activity? Low / Moderate / High

3. On average, how many hours do you sleep at night?

4. Are you concerned about your body weight?

5. Has your oncologist/physician ever discussed the benefits of exercise with you?

Which one: oncologist / primary care / both

6. Do you currently have a membership to a gym or health club? Yes / No

If No: Would you be interested in a membership? No / Not Sure / Why?

7. What are some physical activities that interest you? List all that apply

8. Are you interested in learning more about the benefits associated with exercise and diet? Yes / No Why

9. Have you ever paid a trainer or health professional to help you with exercise or diet? Yes / No

10. On average, how long are you active each day? Minutes / Hours

11. What other types of activities do you normally engage in? List all that apply

12. Do you currently participate in a structured exercise program? Yes / No

If No: a. What limits you (barriers) from being physically active? List all that apply with the things that limit you the most appearing first (prioritize).

b. In the past, have you ever participated in exercise? When / What type / Why did you stop?

c. What environment or setting would you prefer to exercise in?

Home / Gym / Church / Community

d. Would you prefer to exercise with a: Trainer / Group / Alone

If group: Who should be in the group: CRC cancer survivors / Cancer survivors (general) /

Friends / with Family / doesn’t matter

e. Would you prefer: Aerobic / Resistance training / Combination of both

f. Which types of activity would interest you most?

Walking: Treadmill / Outside

Walking on flat surface

Stationary bike

Weightlifting

Yoga

Boot camp

Other:

g. Would you be more likely to exercise if a family member or friend went with you?

If Yes: a. Where do you exercise? Home / Gym / Church / Community group

b. Do you exercise alone or with a partner or group (bootcamp)?

c. What types of exercise do you like to perform? Weights / Treadmill / Bike/ Other

d. How long do you exercise at each session? Minutes

e. How would you describe your level of effort at each session? Low / Moderate / High

f. How many days per week do you typically engage in structured exercise?

g. What motivates you to exercise/stay physically active? List all reasons that apply.

13. Do you have kids? Yes / No

If Yes: a. How many? Boys / Girls

b. Are they physically active? Play structured sports?

c. Do you want them to be physically active?

14. Would weight loss be a reason for you to exercise? Yes / No / Not sure

15. Would you be discouraged if you exercised but did not lose weight quickly? Yes / No / Not Sure

16. What do you feel would be the greatest benefit to a regular exercise program?

17. Are you aware that exercise can reduce cancer risk?

18. Do you live by a park? Would you like to walk / exercise in the park? Why or why not?

19. Do you have a smartphone? Yes/No

If Yes: a. Have you ever heard of or used an exercise or diet app for your smartphone?

Yes / No / If not, would you consider using one?

**Research Participation:**

1. Would you be interested in participating in a research study involving supervised exercise that is of no cost

to you?

If No: a. Why Not?

If Yes: a. How long would you be able or want to commit to participating? Weeks

b. What barriers would stop you from participating? List 3-5 and prioritize them.

c. Where would you prefer to participate in an exercise program?

Private Gym / RPCI / Home / University / Community / Church / Other

d. How many days per week would you be available to participate?

e. How much time would you be willing to spend exercising in each session?

15 min / 20 min / 30 min / 45 min / 60 min

f. What time of day is best? 6-8am / 8-12pm / 12-5pm / 5-8pm

g. Do you prefer weekdays or weekends?

h. Would you prefer sessions to be: Individual / Group / With trainer / No Preference?

i. Would you prefer to exercise with a group of:

CRC cancer survivors / cancer survivors (general) / with a friend/ with family / doesn’t matter

2. Would you prefer: Aerobic exercises / Resistance training / Combination of both

a. Which types of activity would interest you most?

Walking: Treadmill / Outside

Walking on flat surface

Stationary bike

Weightlifting

Yoga

Boot camp

Other

3. Which program schedule would be more likely to commit to?

a. 1 session supervised, 1-2 exercises at home/gym

b. 1 session supervised, 3-4 sessions at home/gym

c. 2 sessions supervised, 2-3 sessions at home/gym

d. 2 sessions supervised, 1-2 sessions at home/gym

e. 3 sessions supervised

d. Other: ________________________________

4. Do you think the exercise program should also include information on nutrition/healthy cooking?

Yes / No / Why / Why Not?

5. Would you attend a lecture on nutrition/healthy cooking? Yes / No

If No: a. Why not?

If Yes: a. Would you prefer evening or weekend?

6. As a childhood cancer survivor, what health topics interest you?

**Demographics:**

1. Race: White/ African American / Asian – other: _______ refused

Ethnicity: Hispanic / Hispanic refused

2. Gender: Male / Female

3. Current Age (years) ____________

4. Year diagnosed _______________ and year completed treatment _______________

5. Treatment: Chemotherapy / Surgery / Radiation (circle all that apply)Chemo

6. Approximate body weight? ____________

7. Approximate height? ____________

8. Marital status: Single / Married / Divorced / Separated / Widowed / Cohabitate

9. Current job: FT / PT / homemaker / unemployed / other: Employer __________________________

SSI / SSD/ both / neither

10. Are you a cigarette smoker? Current / Former / Never

At what age did you start smoking? _____________

At what age did you quit smoking? ____________

Usual number of cigarettes per day: ___________ [1 pack = 20 cigs]

Do you use any other form of tobacco? Pipe / Cigars / Chew / Other

11. Highest level of education

A) High school/GED

B) Technical School

C) Some college

C) Associates degree

D) Bachelor’s degree

E) Graduate degree

12. What was your total household income before taxes during the past 12 months?

A) Less than $25,000

B) $25,000 to $34,999

C) $35,000 to $49,999

D) $50,000 to $74,999

E) $75,000 to $99,999

F) $100,000 to $149,999

G) $150,000 or more

12. Including yourself, how many person reside in your household: ___

Any other comments or thoughts about health and physical activity?

**MEDICAL HISTORY:** Which of the following health issues to you have?

□ history of a heart attack

□ CABG or cardiac stenting or valve surgery

□ congestive heart failure

□ irregular heartbeat

□ chest pain or pressure at rest or with activity

□ diabetes

□ COPD/emphysema

□ high blood pressure

□ chronic low back pain

□ cancer other than Pediatric? : ________________

Last time in hospital (MM/YY) & reason: ______________________________________

**SURGICAL HISTORY:** what surgeries have you had in the past (include year)?

□ __________________________

□ __________________________

□ ___________________________

**ALLERGIES:** list all medication allergies and the effect (e.g., penicillin 🡪 hives/ throating swelling0

**MEDICATIONS:** list all medications that you take on a regular basis, including prescribed and OTC.

Name strength times per day (99=as needed)

**SOCIAL HISTORY:** list all medications

Marital status: single married divorced separated cohabitate

Current job: ______________________________________________ □ FT □ PT

Do you use any other form of tobacco? □ pipe □ cigars □ chew □ other: \

Do you drink alcoholic beverages? ? □ ever □ never

Usual number of drinks per day: ________________ □ “social drinker”

Have you ever been treated for alcoholism/alcohol abuse? □ no □ yes

Have you ever been treated for drug/substance abuse? □ no □ yes

*The last series of question appeals to selected body systems. Tell me which of the following you might have experienced:*

| **section** | **Symptoms** |
| --- | --- |
| Heart | heart attack; palpitations/racing/skipping heart beats, fainting/near fainting, lightheadedness, swelling of hands or feet, leg cramps with exertion, chest pain/discomfort, none |
| Lungs | shortness of breath, asthma, wheezing, cough, excessive phlegm, difficulty breathing when lying flat, none |
| Muscles/ joints | joint pain, joint swelling, stiffness, back pain, muscle aches, arthritis, problems with coordination/balance, none |
| Psych | depression, anxiety, bipolar/manic depressive illness, psychosis, suicidal thoughts, violent thoughts, mood changes, none |
| Endocr | diabetes; excessive urination, excessive thirst, cold intolerance, heat intolerance, none |
